# Bladder Dysfunction and Vesicoureteral Reflux

**DOI:** 10.1155/2008/815472

**Published:** 2008-11-04

**Authors:** Ulla Sillén

**Affiliations:** Pediatric Uronephrologic Center (PUNC), Queen Silvia Children's Hospital, The Sahlgrenska Academy at University of Gothenburg, 416 85 Gothenburg, Sweden

## Abstract

In this overview the influence of functional bladder disturbances and of its treatment on the resolution of vesicoureteral reflux (VUR) in children is discussed. Historically both bladder dysfunction entities, the overactive bladder (OAB) and the dysfunctional voiding (DV), have been described in conjunction with VUR. Treatment of the dysfunction was also considered to influence spontaneous resolution in a positive way. During the last decades, however, papers have been published which could not support these results. Regarding the OAB, a prospective study with treatment of the bladder overactivity with anticholinergics, did not influence spontaneous resolution rate in children with a dysfunction including also the voiding phase, DV and DES (dysfunctional elimination syndrome), most studies indicate a negative influence on the resolution rate of VUR in children, both before and after the age for bladder control, both with and without treatment. However, a couple of uncontrolled studies indicate that there is a high short-term resolution rate after treatment with flow biofeedback. It should be emphasized that the voiding phase dysfunctions (DV and DES) are more severe than the genuine filling phase dysfunction (OAB), with an increased frequency of UTI and renal damage in the former groups. To be able to answer the question if treatment of bladder dysfunction influence the resolution rate of VUR in children, randomized controlled studies must be performed.

## 1. INTRODUCTION

 There is a close relationship between bladder dysfunction and vesicoureteral reflux (VUR). This is most evident in children with neurogenic bladder dysfunction, in which the high intravesical pressure due to outflow obstruction induced by detrusor/sphincter dyssynergia is directly responsible for the development of the reflux. Another example of VUR as a secondary phenomenon is in boys with posterior urethral valves, where the reflux is secondary to an anatomical obstruction in the urethra. In most cases of secondary reflux, normalisation of bladder function means spontaneous resolution of the VUR.

 When it comes to primary VUR, a close connection to bladder function pathology of nonneurogenic origin has also been established. Studies have been published describing children with functional bladder disturbance and VUR after toilet-training age. Studies from the 1980s most often dealt with girls who have overactive bladders (OAB) in combination with reflux, and treatment of the dysfunction positively influenced resolution of the reflux. During the last decades, however, bladder dysfunction including the voiding phase, such as dysfunctional voiding and dysfunctional elimination syndrome, has been reported to have a negative influence on VUR resolution in some studies. In other studies, treatment of the dysfunction has improved the resolution rate.

 Children with high-grade congenital reflux have also been shown to have abnormal bladder function in about half of the cases. This dysfunction was characterised by an overdistended bladder and incomplete emptying. The dysfunction per se had a negative influence on spontaneous resolution of VUR, which did not improve despite treatment.

In this overview of bladder dysfunction and VUR, only primary reflux is discussed. There are some contradictory results in the literature available, as indicated above, which make an overview interesting. One of the major problems is the fact that the level of evidence in almost all papers is low, most have level three, a few have level two, and no paper was identified as level one.

## 2. STANDARDISATION OF TERMINOLOGY

When studying the literature about bladder dysfunction in refluxing children, one finds that there is still confusion emanating from differences in terminology (especially between the US and Europe), diagnostic procedures (urodynamic or clinical), degree of dysfunction, and so on. In this review, two bladder dysfunction entities are addressed in children after the age for bladder control: overactive bladder (OAB) and dysfunctional voiding (DV). The ICCS definitions of these entities are used. This means that dysfunctional voiding is only used in the sense of a dysfunction during the voiding phase, characterised by increased activity in the pelvic floor during voiding. The term can only be applied if repeat uroflow measurements show a staccato or interrupted pattern [1]. Dysfunction elimination syndrome (DES) is also discussed, and refers to an abnormal pattern of both bladder and bowel. It is characterised by withholding, often with incontinence. The bladder part of this syndrome can be recognised as dysfunctional voiding.

The overall term recommended in the standardisation document for bladder dysfunction is lower urinary tract (LUT) dysfunction. This term is used as a synonym for bladder dysfunction in the present overview.

## 3. BLADDER DYSFUNCTION CHARACTERISTICS IN CHILDREN WITH VUR

### 3.1. Before the age for bladder control

In the early 1990s, bladder dysfunction was reported in small infants with high-grade reflux. The dysfunction was characterised by low bladder capacity, high voiding pressure, and overactivity during filling [2–4]. Later studies have shown that both low capacity and high voiding pressure are normal findings in urodynamic investigations in this age group [5], while longitudinal studies of children with high-grade VUR diagnosed during infancy have shown a completely different bladder function pattern after the infant year; high capacity bladder with incomplete voiding [6, 7].

Dyscoordination at voiding was often seen in these young children with VUR, a finding that can be suggested as the reason for the high capacity bladder with incomplete emptying.

However, investigations of nonrefluxing children have shown that dyscoordination at voiding was seen in healthy infants, both in cystometric [5] and free voiding studies [8]. The free voiding studies are longitudinal investigations in healthy children and the finding was recognised as an immature phenomenon that was quite common early in infancy and then decreased and was not seen after the age for bladder control [8].

Interestingly, a bladder dysfunction pattern that was similar to the above-mentioned high capacity bladder with incomplete emptying dynamics was described as early as 1987 by Griffiths and Scholtmeijer [9] in urodynamic investigations of young children with VUR. They showed two distinct bladder patterns. The most common (about 50%) in the 104 VUR patients was characterised by urethral overactivity during voiding with relative weak voiding contractions, while detrusor overactivity during filling was not a major finding. This bladder pattern was seldom seen together with incontinence, but the reflux was often bilateral, combined with renal abnormalities and frequent UTIs, findings similar to what have been shown for the children, discussed above, with congenital high-grade VUR [6, 10]. The other bladder pattern, seen in about 25%, was urodynamically characterised by overactivity often seen together with bladder symptoms such as incontinence. The kidneys were often normal, the VUR often unilateral, and UTIs were seldom seen.

High-capacity bladder has also been reported in other studies in children with VUR after the infant year, especially in boys [11] and together with dilating VUR [11–13]. It has also been reported as a factor that is negatively correlated to spontaneous resolution of the reflux.

### 3.2. After the age for bladder control

**Prevalence of LUT dysfunction in children with VUR**

The reported prevalence of bladder dysfunction in a VUR population varies. When diagnosed using invasive urodynamic investigations, higher figures were generally found (38%–75%) in contrast to what was seen when nonurodynamic investigations were used (18%–52%) (Table 1). This variation was probably related to factors such as grade of VUR, age of the children, and, obviously, how the dysfunction was diagnosed.

The earliest studies of bladder dysfunction mostly dealt either with dysfunctional voiding or the overactive bladder. Recent studies often give the prevalence of both dysfunctions together in children with VUR. The advantages of separating the dysfunctions are that different treatment modalities can be evaluated, as well as differences in results when it comes to effects on VUR resolution. The disadvantage is that the dysfunctions often are combined and sometimes difficult to separate.

The first reports about bladder dysfunction and VUR came in the 1970s. Hinman and Baumann [22] and Allen [23] described a severe form of dysfunctional voiding that was suggested to cause the VUR in parallel to what was seen in neurogenic bladder dysfunction. The investigations indicated that the condition was rarely seen, but no prevalence figures were reported.

A few years later, Koff and Murtagh [18] and Taylor et al. [17] reported on OAB in conjunction with VUR, and they found high prevalence figures for the dysfunction (55–75%), mainly seen in girls after the age for bladder control. Koff and coworkers [18] also indicated that the reflux had a higher spontaneous resolution rate after treatment of the bladder problem with bladder regimen and anticholinergics, as compared with a group with similar grades of reflux but without overactivity in the bladder (Table 3). Other more recent studies (Table 1) showed a prevalence of OAB between 25% and 38% in urodynamic studies of children with VUR of different grades, whereas the prevalence in a nonurodynamic study was only 8% (Table 1).

In studies of larger cohorts of children with VUR, the prevalence of all bladder dysfunction together was reported to be between 18% and 50%, using questionnaires and flow measurements for the diagnose [12, 14, 16]. In one of the studies, the international reflux study in children [12], differentiation of the dysfunction entities was done and they were found to be almost equally common (Table 1). This latter relation was also seen in urodynamic investigations [9, 20, 21], although the total number of children with dysfunction was higher in those studies (Table 1). This relation between OAB and dysfunctional voiding is very different from what is considered to be the case in cohorts of children with voiding dysfunction without VUR, in which OAB is much more common than dysfunctional voiding, especially in nonurodynamic studies [24] but even in urodynamic studies [25].

The concept dysfunctional elimination syndrome (DES) was introduced by Koff et al. in 1998 [20], including infrequent voiding, constipation, and often symptoms of an overactive detrusor. He reported it to be present in 46% of children with primary reflux (Table 1). He found that both the rate of UTI and spontaneous resolution of VUR were adversely influenced by the presence of dysfunctional elimination syndrome. He also noted that in the children who had detrusor overactivity as their main dysfunction, the likelihood of recurrent UTI was lowest, indicating that the OAB dysfunction was less severe.

These latter results were in line with what was seen in a followup study at the age of 7 years of 20 children who presented during infancy with grade 4-5 VUR and bladder dysfunction, diagnosed at that time. The dysfunction was characterised by high bladder capacity and incomplete emptying. At the followup, these children had infrequent voiding, and often did not void at school or in the morning if not prompted by parent or other guardian. Constipation had been or was still a problem in the majority of these children [26]. The reported bladder and bowel dysfunction in these children with congenital reflux was very similar to the DES children as reported by Koff et al. [20]. In these cases, DES actually seems to be a part of the VUR complex and present already from infancy, and might even be suggested to be a congenital problem, rather than an acquired one.

However, in other studies it has not been possible to diagnose dysfunctional elimination syndrome more often in children with VUR than in control groups. Shaikh et al. [27] investigated the prevalence of DES at school age, in a cohort of children with a history of UTI before the age of 2 years. They had a control group of similar age but without a history of UTI. DES was diagnosed in 20% of the children in both groups. In the UTI group, the prevalence of DES did not differ in children with and without VUR, identified earlier in life. The authors conclude that neither UTI nor VUR diagnosed in early childhood was associated with an increased likelihood of DES later in life. Similar results were found in multivariate analyses of a large pediatric patient database with the aim of describing the relationship between DES, sex, VUR, and UTI [28]. Of the total number of patients (2759), about two-thirds had VUR. DES was seen in 35% overall, with the highest prevalence in patients without VUR but with a history of UTI (52%). The lowest frequency was found in VUR without UTI (22%), whereas in those with VUR and UTI it was 39%. Thus DES was less common in VUR children than in children without VUR, especially if not found together with UTI. Another important finding was that girls had a significantly higher rate of DES than boys.

**Prevalence of VUR in children with bladder dysfunction**

In studies where the inclusion criterion was idiopathic bladder dysfunction, the prevalence of VUR was between 14% and 47% [25, 29, 30] (Table 2). The variation was probably attributable to selection of patients referred to the unit. The lowest frequency was found in a urodynamic study of 1000 consecutive children referred to a large urotherapy unit. In this latter study, less selection of patients can be suggested than in the other studies cited, since in these other studies the patients were referred to a pediatric urological clinic.

## 4. TREATMENT OF VOIDING LUT DYSFUNCTION AND ITS EFFECTS ON VUR IN CHILDREN WITH BLADDER CONTROL

The registration of severity of voiding LUT dysfunction and its response to treatment with regard to symptoms is often highly subjective, since the definition of how often the symptoms are experienced is seldom given. Furthermore, in most cases a number of symptoms are included. Using a symptom score has been suggested in order to overcome this obstacle. Upadhyay and coworkers [31] reported on a group of children with both bladder dysfunction and VUR using symptom score to evaluate severity of bladder dysfunction before any treatment and also to record the results after treatment at followup. Overall after 2 years, resolution and downgrading of VUR was 58%, with a decrease in symptom score from 9.6 to 3.7 in this group. In the group without improvement of the reflux, on the other hand, the symptom score went from 14.4 to 11.1, that is, a higher initial score and also poor response to the treatment. The weakness of the scoring system is that all symptoms have the same value, no symptom is considered more serious than another.

**Overactive bladder**

The results of treatment of overactivity in relation to VUR resolution are conflicting. Most studies do not have a control group, include only a small number of patients, are retrospective, and have nonuniform ways of diagnosing overactivity, which might explain the different results. Many studies suggest increased spontaneous resolution after such treatment [18, 32, 33] (Table 3). As early as 1983, Koff and Murtagh [18] reported that anticholinergic treatment of detrusor overactivity in 26 girls gave a VUR resolution rate of 44% during a 4 year followup, as compared with a group of children with VUR but without detrusor overactivity, in which the resolution was only 17%. In a similar comparison, Scholtmeijer and Nijman [19] found only a slightly higher rate of improved grade of VUR in the group treated for detrusor overactivity (Table 3).

Conversely, Willemsen and Nijman [34] showed, in a prospective study of 102 children, that treatment of the group with detrusor overactivity (41 children, 40%) with anticholinergic drugs did not increase their resolution rate, as compared with a group without overactivity. The overall resolution rates were 51% and 55%, respectively for those with and without overactivity (Table 3). An increased rate of UTI, however, was found in the children with bladder overactivity.

Whether spontaneous resolution of VUR in a group with untreated OAB is different from a group without OAB cannot be established from the studies available.

**Dysfunctional voiding (DV)**

There are very few studies reporting on VUR and treatment of isolated DV, while there are more on DV and detrusor overactivity seen together.

Kibar et al. [36] reported on treatment with biofeedback in children with DV and VUR. The overall resolution rate after less than one year of followup was 63% (Table 3). No controls were used. Similar results was reported by Palmer et al. [35], with resolution in 55% and downgrading in 16% of VUR one year after biofeedback treatment of DV (Table 3). Grades of VUR were mainly I-III in the latter study, while in the former some grade IV were also included.

Homsy et al. [32] reported as early as 1985 that treatment of bladder dysfunction (overactivity only or together with dysfunctional voiding) with anticholinergics influenced the spontaneous resolution rate of VUR. He noted that a small subgroup of children without incontinence had a VUR resolution of only 6%, whereas in those with urinary incontinence the resolution rate was 68% during 2.5 years of treatment and followup.

Snodgrass [15] noted a lower resolution rate of VUR in children with dysfunction. The problem with the presentation of this cohort of children with VUR and bladder dysfunction was that OAB and dysfunctional voiding were not differentiated. This is a problem when it comes to treatment with oxybutynine. This treatment may be contraindicated in dysfunctional voiding because of incomplete emptying before start of treatment, thus inducing higher risk for UTI, which was seen in his series.

However, including studies in which bladder dysfunction was characterised by a dysfunctional voiding pattern, data support the assumption that there is a decreased spontaneous resolution of VUR in children with this dysfunction, especially when seen in combination with high-grade VUR.

Yeung et al. [21] showed, in children between ages one and eleven, that bladder dysfunction and renal abnormalities were significant negative prognostic factors for resolution. He did not report on any treatment or its possible treatment effects on this rate. The same finding was established in the IRSC study [12], that is, children with bladder dysfunction had a lower resolution rate of reflux.

DES in children with VUR was also correlated to a lower resolution rate [20], despite treatment of both the bladder and bowel dysfunction. Similar results have been reported in studies before the age for bladder control. In these studies, the dysfunction was characterised by high capacity bladder and incomplete voiding [6, 10]. In a study where this kind of dysfunction was diagnosed before the age for bladder control, treatment with clean intermittent catheterisation did not increase the spontaneous resolution rate in 20 children with grade 4-5 VUR [37].

## 5. BLADDER DYSFUNCTION AND RESULTS OF SURGICAL/ENDOSCOPIC VUR TREATMENT

It has previously been suggested that reimplantation of the ureter into the bladder in a child with major voiding dysfunction carries a high risk of failure. The dysfunction that carries the high risk is a severe form of dysfunctional voiding, induced by functional obstruction during voiding [22, 38]. Regarding endoscopic VUR treatment, milder forms of voiding LUT dysfunction did not influence the results of endoscopic injection treatment for VUR in a recent study [13], in which the dysfunction disappeared after cessation of the reflux. The authors suggest that the reflux was an underlying cause of the dysfunction in these cases. Additionally, they observed that a high proportion of those requiring a second injection had persistent bladder dysfunction of a different kind, characterised by high bladder capacity and infrequent voiding. This again suggests that the dysfunctional bladder, but not the isolated OAB, is a risk for failure of active reflux treatment. Another study reported that the success rate was lower after a second injection in children with bladder dysfunction [39]. In this study, the type of dysfunction was not specified.

## 6. UTI, BLADDER DYSFUNCTION, AND VUR

Recurrent UTIs have been shown in many studies to be higher in VUR patients with bladder dysfunction than in VUR children without such dysfunction [6, 15, 20]. This was most obvious in children with emptying problems such as in DV and DES as well as in children with congenital high-grade reflux and incomplete emptying.

Snodgrass [15] showed a higher frequency of UTI in children with VUR who also had bladder dysfunction. The dysfunction was treated with oxybutynine in all cases. The problem with the presentation of this cohort of children with VUR and LUT dysfunction was that OAB and dysfunctional voiding were not differentiated. This is a problem when it comes to the treatment with oxybutynine, since it may be contraindicated in dysfunctional voiding because of incomplete emptying before starting treatment, thus inducing higher risk for UTI.

## 7. RENAL SCARRING, BLADDER DYSFUNCTION, AND VUR

Most of the studies reporting on children with VUR and the OAB dysfunction have not found any difference in numbers of children with renal damage in the groups with and without the dysfunction [17, 18]. In a study including a small number of patients, however, a slightly higher number of damaged kidneys were seen in children with VUR and OAB [40]. On the other hand, differences between those with DV and the OAB dysfunction have been identified, with higher frequency of renal damage in children with DV [9].

## 8. CAUSAL CONNECTION BETWEEN VUR AND BLADDER DYSFUNCTION

The bladder function pattern with high capacity bladders and incomplete emptying seen at follow up in children presenting during the infant year with high-grade VUR [6, 10] is similar to the dysfunctional voiding pattern seen in older children [9, 12]. The majority of children with congenital high-grade VUR have been reported to have recurrent UTI and renal damage, as well as poor spontaneous resolution of the reflux [6, 10], which is also similar to what has been reported for older children with VUR and dysfunctional voiding [9, 12, 15]. The dysfunctional voiding can be suggested to be a milder form of the Hinman bladder [22]. In the Hinman bladder, the dysfunction is thought to be the primary problem and acquired after age for bladder control, and the cause of VUR. In the congenital high-grade VUR, on the other hand, both the dysfunction and the VUR may be congenital. Actually, a common cause of the reflux, the bladder dysfunction and the general hypo/dysplasia often seen in the ipsilateral kidney, can be suggested. An anomaly in the ureteric bud region could be suggested to induce the VUR and the renal anomaly. Since these embryological structures also form the bladder outlet, the dysfunction of the bladder might theoretically also have the same origin [41]. A more severe form of congenital dyscoordination, than the physiological, is another possibility.

The extra volume load induced by the refluxing urine volumes, which circulate between the bladder and the upper urinary tract, might also be a factor of importance for the high capacity bladder. In such cases, the bladder problems should more or less disappear after surgical treatment of the reflux. Investigation of bladder function in a group of children ages 7-8 years who had been surgically treated for high-grade reflux at the age of median 4 years did not support this theory. These children were diagnosed early as having a bladder dysfunction characterised by high-capacity bladders with incomplete emptying. At the follow-up investigation, they still had high capacity bladders with few voiding per day but their emptying ability had improved, with quite low volumes of residual urine [26]. The results of this study did not support the theory of the refluxing volumes as a cause of the high capacity bladder.

The connection between the overactive bladder dysfunction pattern and reflux is less clear. It is difficult to consider bladder overactivity the cause of reflux, since it causes only intermittent increases in bladder pressure, which is not thought to induce reflux if the junction is competent. Only a concomitant obstruction inducing a continuous pressure problem in the bladder is considered to be able to induce VUR in parallel to what is seen in children with the NBD or anatomical urethral obstruction, for example, in boys with PUV. The other possibility is that there is only marginal competence in the valve mechanism, and in these cases the detrusor contractions against a contracted sphincter may induce VUR. If this latter causality exists, it might explain why renal damage seldom is seen in children with an OAB [9]: the pressure influencing the kidneys is only intermittent. Furthermore, these children are often recognised after toilet training age, that is, VUR is not congenital but occurs when the kidneys can be suggested to be less vulnerable. In addition, VUR is often of low grade. A few studies have shown a similar number of patients with renal abnormalities both in groups with bladder overactivity and in groups with stable bladder [17, 18]. In these studies, the control group was, however, children with VUR but without any bladder dysfunction.

## 9. COMMENTS

VUR is associated with both OAB and dysfunctional voiding, with different entities as described above. However, we can only speculate about the precise causative mechanisms between the respective dysfunction and VUR. There are divergent opinions concerning whether the treatment of the overactive bladder influences the rate of spontaneous resolution. There are as yet no randomised studies investigating the effect on the reflux of treatment of the OAB versus no treatment. To my knowledge, there are no studies comparing a group of children with VUR and untreated OAB with a group of children with VUR and a stable bladder.

In children with dysfunctional voiding and VUR, it is easier to see a causative connection, especially in the more severe forms of VUR, since this can be considered parallel to neurogenic bladder dysfunction. It is not known whether this is an acquired dysfunction as most authors suggest or if it is a congenital anomaly and part of a complex that also includes VUR.

Treatment of the dysfunctional voiding increases the spontaneous resolution rate as has been suggested in some studies, but not in others. Since there are no randomised studies available comparing resolution rates in treated children with untreated children, this cannot be established. However, what is known is that dysfunctional voiding, dysfunction elimination syndrome, and the similar dysfunction seen in children with high-grade congenital reflux, all have negative influences on the spontaneous resolution rate of VUR when untreated, and lead to an increased risk for recurrent UTI.

Since there seems to be a lower resolution rate in children with dysfunctional voiding than in those with OAB, it is important to distinguish between the diagnoses when comparing VUR resolution rates of children with and without dysfunction. OAB in its genuine form seems to be a much more benign dysfunction than dysfunctions including incomplete emptying of the bladder. However, it should be remembered that bladder overactivity and dysfunctional voiding are often seen together.

In summary, the question if treatment of bladder dysfunction improves prognosis for spontaneous resolution of reflux cannot be answered from the studies available. This is true for the overactive bladder, the dysfunctional voiding, as well as the dysfunctional elimination syndrome. Randomised studies have to be performed to give an answer. In these studies also the definitions from the ICCS standardisation document have to be used, to avoid confusion about terminology. Maybe the use of a scoring system of bladder dysfunction symptoms would be useful as well. However, treatment of bladder dysfunction should of course be recommended, especially in cases with dysfunctional voiding and DES. One reason is that the success of surgical treatment of the reflux, both endoscopic and open, probably depends on the bladder function status. The most obvious reason for treating the bladder dysfunction in these refluxing children is, of course, as in nonrefluxing children, the symptoms of urgency, urinary incontinence, constipation, UTI, and so on.

## Figures and Tables

**Table 1:** Prevalence of bladder dysfunction in patients with VUR.

Reference	Age (years)	Patients with VUR (number)	Bladder dysfunction (% of total)	Overactive bladder (% of total)	Dysfunctional voiding (% of total)	Dysfunctional elimination syndrome (% of total)
*Nonurodynamic investigations*						
Snodgrass 1991 [14]	0.1/16	39	20%			
Van Gool et al. 1992 [12]		310	18%	8%	6%	
Snodgrass 1998 [15]	3–10	128	52%			
Homayoon et al. 2005 [16]	>3.5–4	342	20%			

*Urodynamic investigations*						
Taylor et al. 1982 [17]	4–15	37	75%	75%		
Koff & Murtagh 1983 [18]	2–14	62	55%	55%		
Griffiths & Scholtmeijer 1987 [9]	2–15	104		**25% (23%)	**14% (25%)	
Scholtmeijer & Nijman 1994 [19]	0.1–15	101	38%	38%		
Koff et al. 1998 [20]	after bladder control	143	46%	27%	23%	46%
Yeung et al., 2006 [21]	1–11	82	55%	*38	*27

*% of those with bladder dysfunction, **in brackets additional number with OAB and dysfunctional voiding, respectively, but with some uncertainty of the diagnosis.

**Table 2:** Prevalence of VUR in patients with bladder dysfunction. Urodynamic studies.

Reference	Age (years)	Patients with bladder dysfunction (number)	Overactive bladder (% of patients with VUR)	Dysfunctional voiding (% of patients with VUR)	Patients with VUR (% of total)
*Koff et al. 1979 [30]	2.5–17	53	100%		47%
Hoebeke et al. 2001 [25]	9–10	1000	58%	31%	14%
Ural et al. 2008 [29]	1.5–15	340	71%	6%	46%

*Only patients with UTI included.

**Table 3:** Impact of treatment of bladder dysfunction on spontaneous resolution of VUR.

Reference	Age (y)	Patients (number)	VUR grade	Bladder dysfunction	Treatment	Follow/up (y)	Resolution (downgrading)	Controls resolution (downgrading)
Koff & Murtagh 1983 [18]	2–14	62	I-IV	OAB	Anticholonergics	4	44% (16%)	17% (0%)
Scholtmeijer & Griffiths 1990 [33]		25	I-IV	OAB	Anticholinergics	1	37% (22%)	No controls
Scholtmeijer & Nijman 1994 [19]	0.1–15	39	I-IV	OAB	Anti-cholinergics	3	38% (38%)	40% (16%)
Willemsen & Nijman 2000 [34]	0.1–15	102	I-V	OAB	Anti-cholinergics	5	51%	55%
Palmer et al. 2002 [35]	6–10	25	I-III	DV	Biofeedback	1	55% (16%)	No controls
Kibar et al. 2007 [36]	7.2	78	I-IV	DV	Biofeedback	0.5	63% (29%)	No controls
Homsy 1985 [32]	4–11	35	I-IV	OAB + DV	Oxybutynine	2.5	50% (22%)	No controls
Snodgrass 1998 [15]	3–10	128		OAB + DV	Oxybutynine		45%	61%
